# Supplementary material for: M72 Fusion Proteins in Nanocapsules Enhance BCG Efficacy Against Bovine Tuberculosis in a Mouse Model
Source: Pathogens. 2025 Jun 16;14(6):592. doi: 10.3390/pathogens14060592 (PMC12195942; doi:10.3390/pathogens14060592)
Supplement: Supplementary file 1 [file pathogens-14-00592-s001.zip › Supplementary material/Figure S1.pdf]

Figure S1: Characterization of NCs

I. SEM images and size distribution histograms measured using DLS of NCs: a) unloaded CS/ALG NCs, b) CS/ALG NCs antigen-loaded M72, c) CS/ALG NCs antigen-loaded ABDsM72. The results are outlined in the table at the bottom of the image.

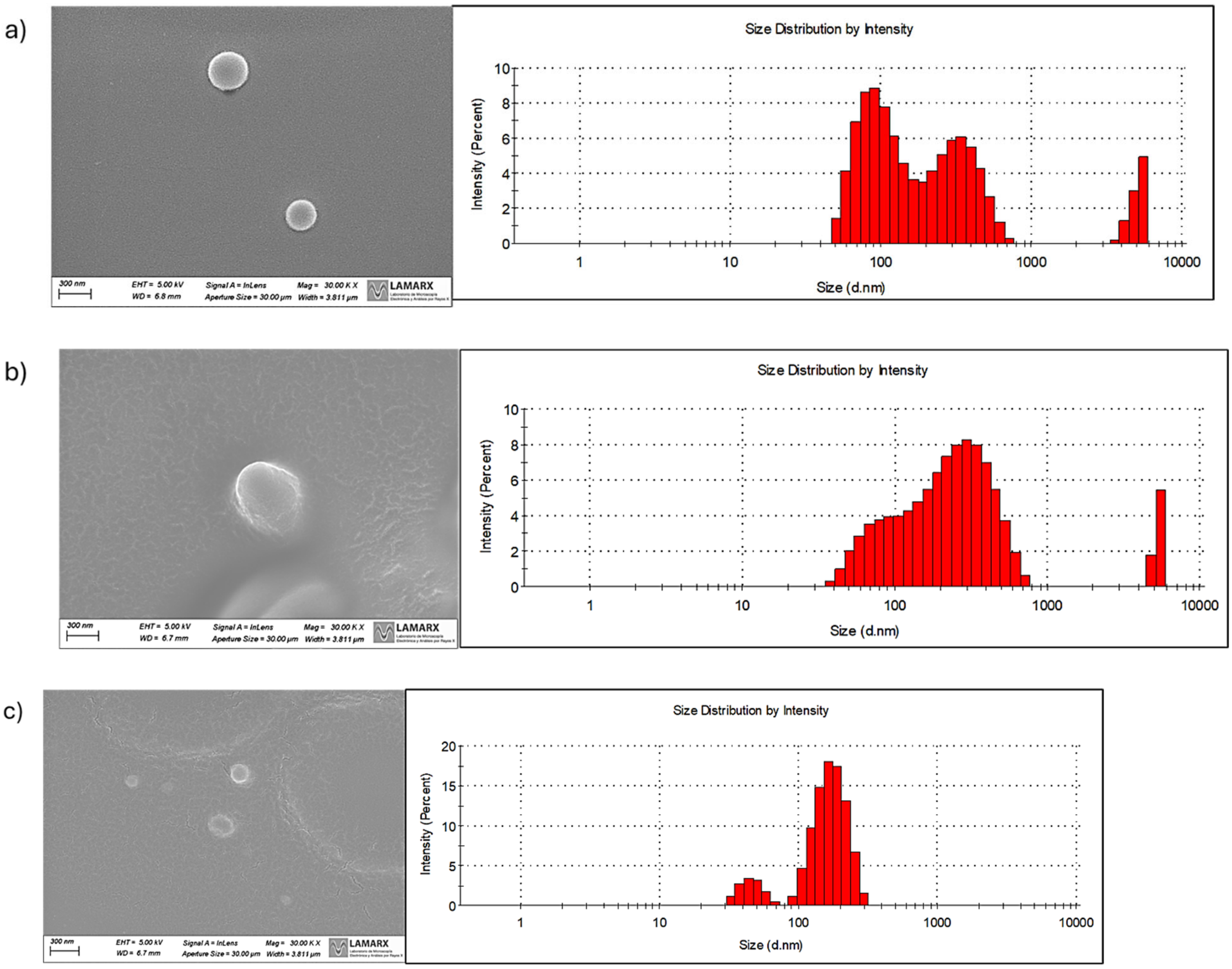

II. Hydrodynamic radius and z-potential by DLS of the NCs

Samples	Size (nm) by SEM	Size (nm) by DLS	Pot Z (mV) by DLS
CS/ALG NCs - Freeze-dried	164 ± 4	103 ± 37	-37 ± 1
CS/ALG-loaded M72 NCs - Freeze-dried	172 ± 3	245 ± 47	-25 ± 1
CS/ALG-loaded M72-ABD NCs - Freeze-dried	168 ± 6	175 ± 44	-28 ± 2

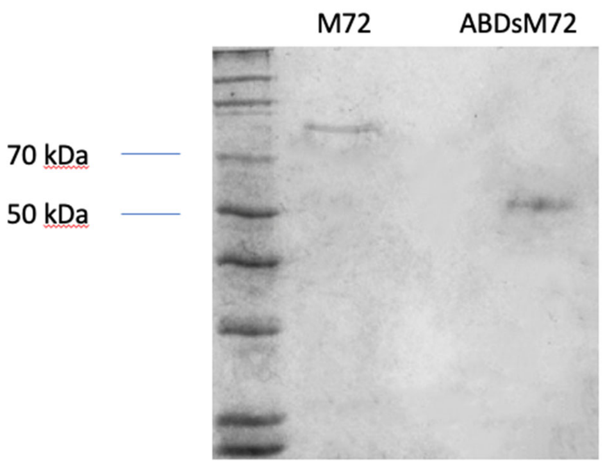

III. Recombinant proteins loaded into NCs. Five microliters of the protein-NC formulation were resuspended in loading buffer, separated by 12% SDS-PAGE, and stained with Coomassie Brilliant Blue.
