# Supplementary material for: M72 Fusion Proteins in Nanocapsules Enhance BCG Efficacy Against Bovine Tuberculosis in a Mouse Model
Source: Pathogens. 2025 Jun 16;14(6):592. doi: 10.3390/pathogens14060592 (PMC12195942; doi:10.3390/pathogens14060592)
Supplement: Supplementary file 1 [file pathogens-14-00592-s001.zip › Supplementary material/Table S1 .pdf]

Table S1. primers used in this study

Name	5´-3´sequence	Region amplified
UpAminoM72<br>LowAminoM72	GAATTCGCCGCCACCATGGCATTGAACGGGTTGAT<br>GAATTCGGATCCGTTAAGCGCGTCCGCCATCG	5´M72
UpmedioM72<br>LowMedioM72	GGATCCATGGTGGATTTCGGGGCGTT<br>GGATCCAAGCTTGCCGGCCGCCGGAGAATGCG	Middle M72
UpCarboxM72<br>LowCarboxM72	AAGCTTATGAGCAATTCGCGCCGCCG<br>AAGCTTGGTACCTCACCCGTTCAATGTCTCTT	3´M72
