# Supplementary material for: M72 Fusion Proteins in Nanocapsules Enhance BCG Efficacy Against Bovine Tuberculosis in a Mouse Model
Source: Pathogens. 2025 Jun 16;14(6):592. doi: 10.3390/pathogens14060592 (PMC12195942; doi:10.3390/pathogens14060592)
Supplement: Supplementary file 1 [file pathogens-14-00592-s001.zip › Supplementary material/Table S2.pdf]

Table S2. Histopathology in mouse organs

Group	Peribronchial BALT	Congestion in liver	Lesion in spleen	ZN+
BCG	7/8	8/8	NL	0/8
BCG+M72	8/8	6/8	NL	0/8
BCG+ABDsM72	6/8	8/8	NL	0/8
PBS	6/7	878	NL	0/7

BALT: bronchus-associated lymphoid tissue; NL: no lesions. ZN+: Ziehl-Neelsen positive bacillus.
Positive samples/total samples
