# Supplementary figures and images for: M72 Fusion Proteins in Nanocapsules Enhance BCG Efficacy Against Bovine Tuberculosis in a Mouse Model
Source: Pathogens. 2025 Jun 16;14(6):592. doi: 10.3390/pathogens14060592 (PMC12195942; doi:10.3390/pathogens14060592)

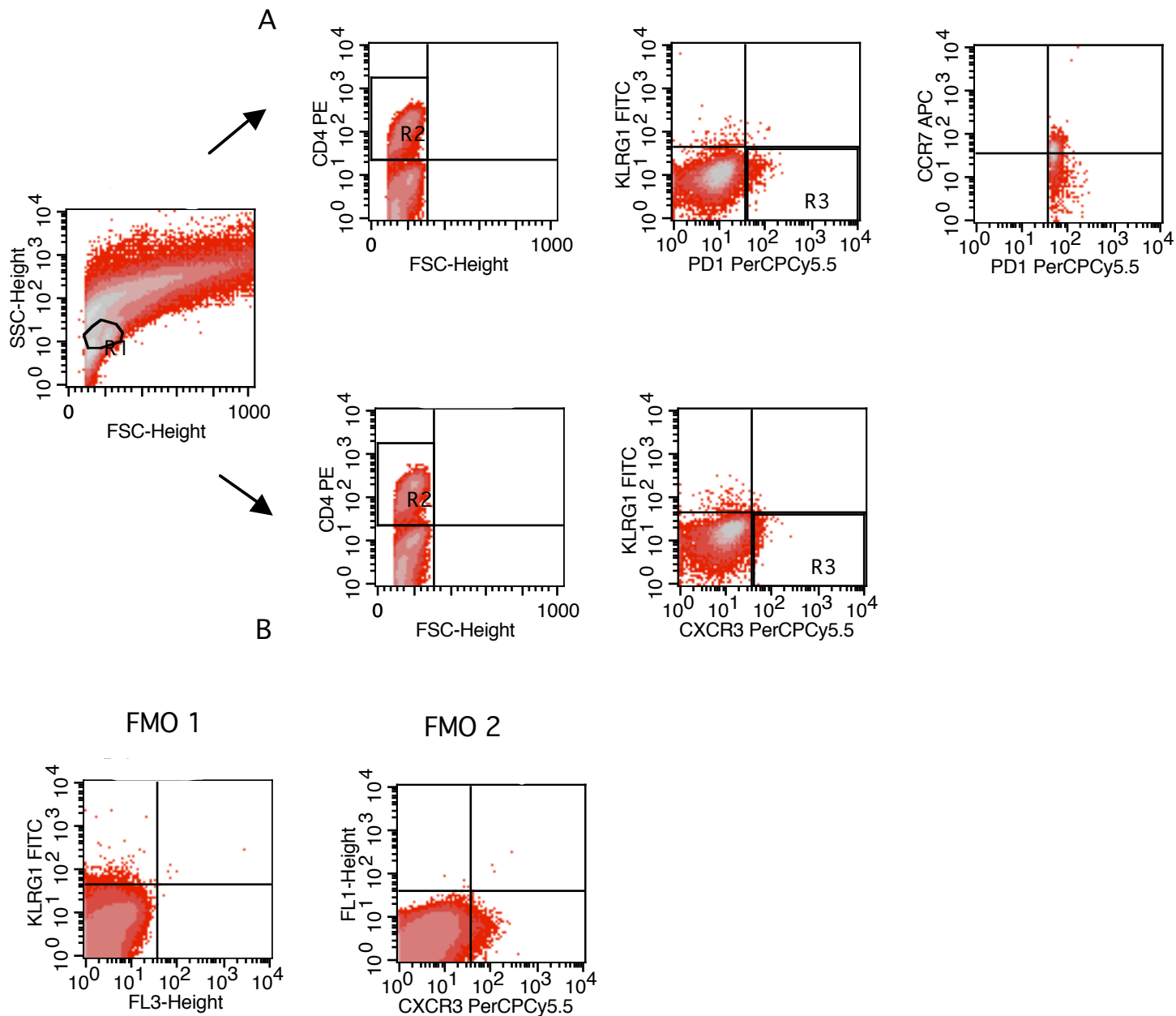

Supplement: Supplementary file 1 [file pathogens-14-00592-s001.zip › Supplementary material/Figure S2A.pdf]

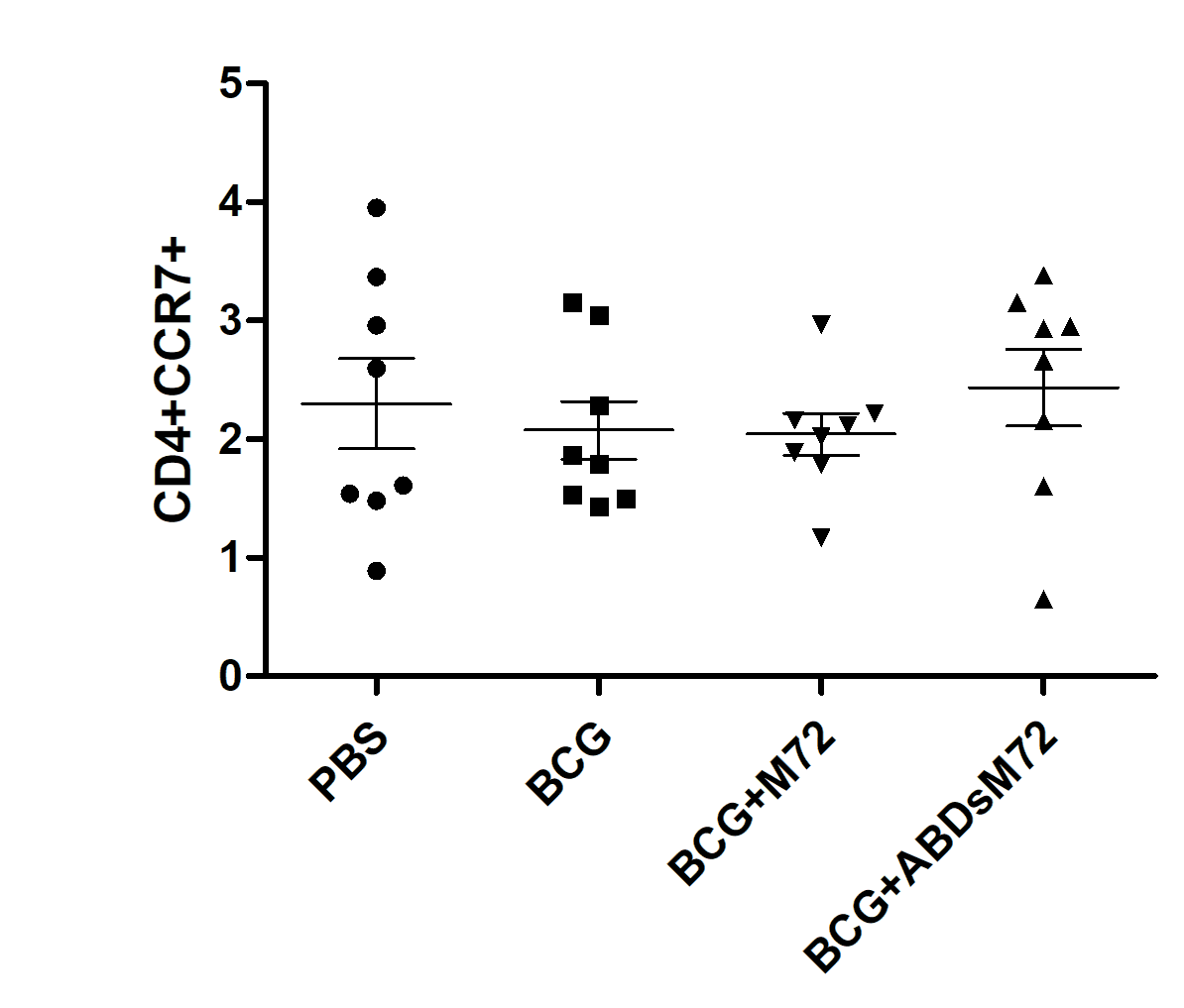

Supplement: Supplementary file 1 [file pathogens-14-00592-s001.zip › Supplementary material/Figure S2B.tif]

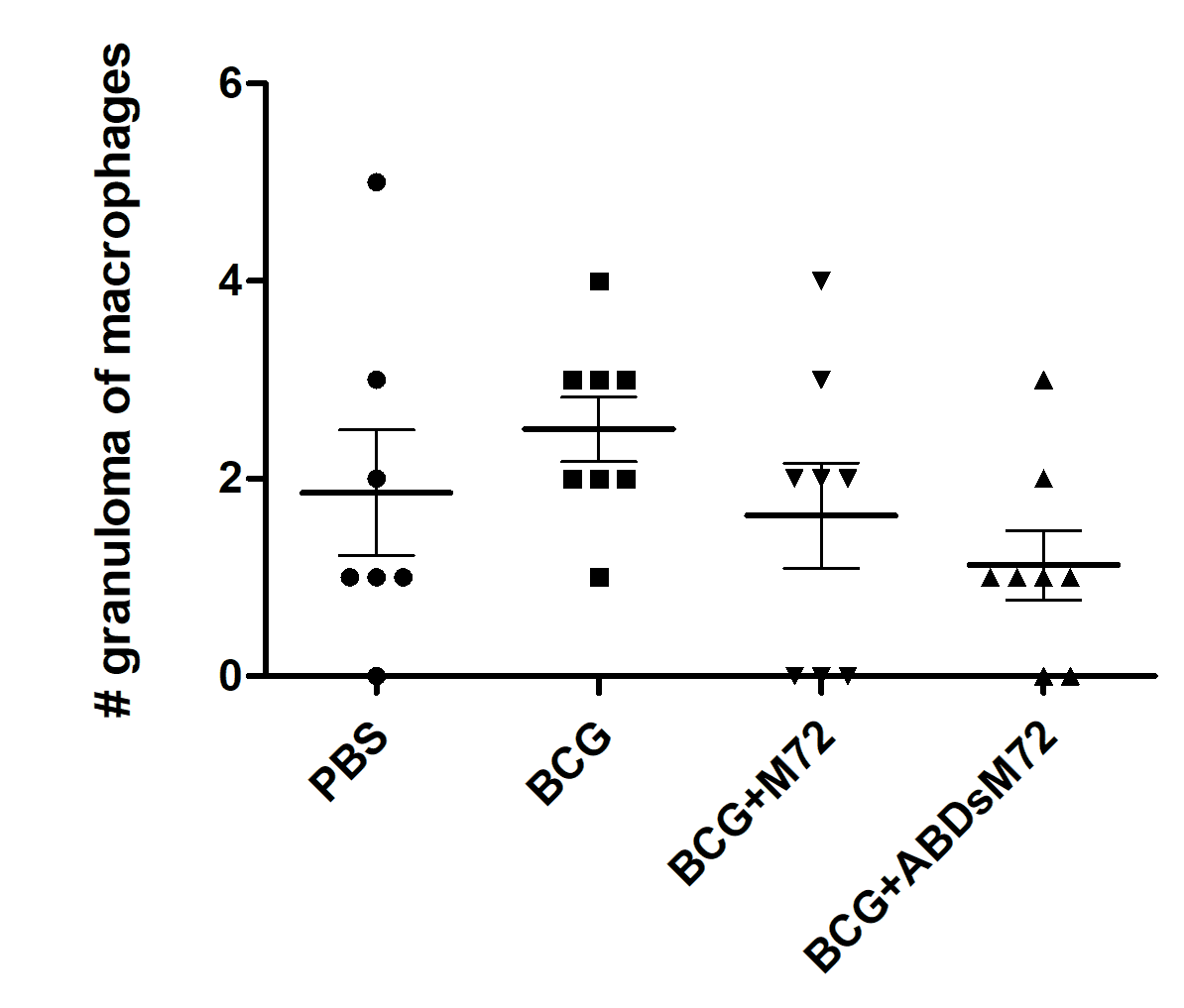

Supplement: Supplementary file 1 [file pathogens-14-00592-s001.zip › Supplementary material/Figure S3.tif]

## Slide 1
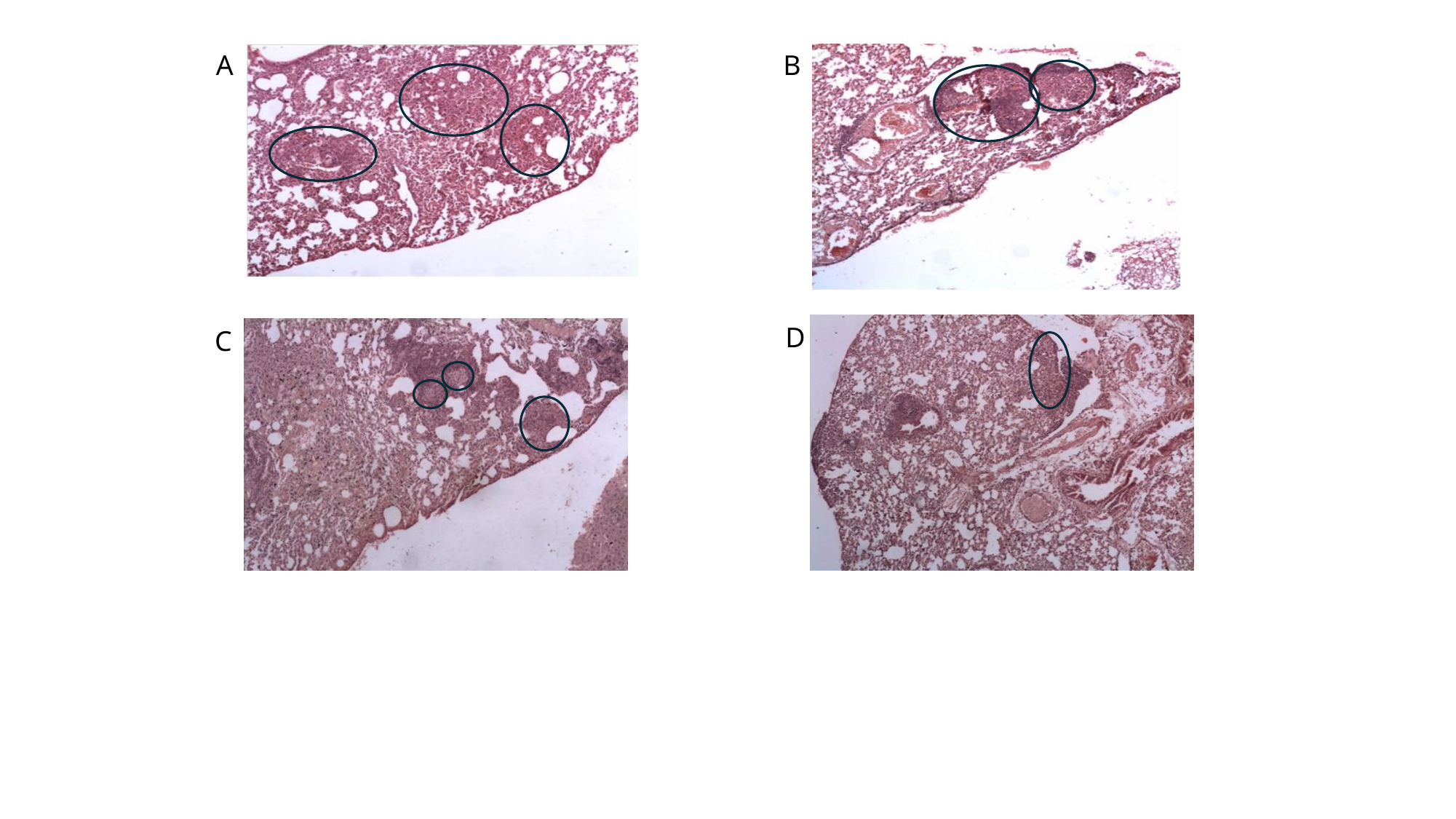

A
B
D
C

Supplement: Supplementary file 1 [file pathogens-14-00592-s001.zip › Supplementary material/Figure S4.pptx]

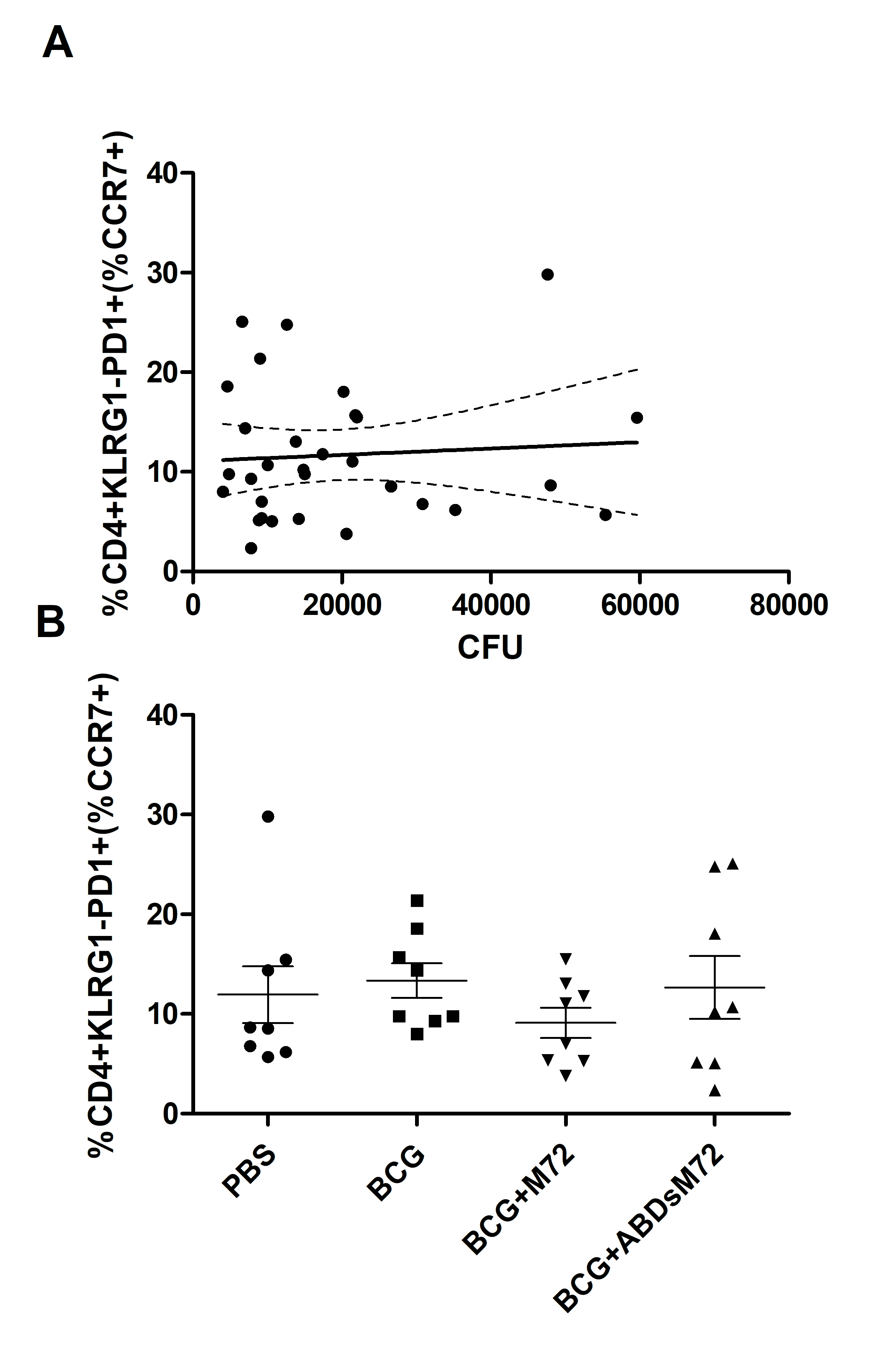

Supplement: Supplementary file 1 [file pathogens-14-00592-s001.zip › Supplementary material/Figure S5A and B.tif]
